# Is Breast Size Related to Prevalent Thoracic Vertebral Fracture? A Cross‐Sectional Study

**DOI:** 10.1002/jbm4.10371

**Published:** 2020-05-19

**Authors:** Linda Spencer, Leanda McKenna, Robyn Fary, Richard Ho, Kathy Briffa

**Affiliations:** ^1^ School of Physiotherapy and Exercise Science Curtin University Perth Western Australia Australia; ^2^ Perth Radiological Clinic Perth Western Australia Australia

**Keywords:** BIOMECHANICS, FRACTURE RISK ASSESSMENT, DXA, MENOPAUSE

## Abstract

Large breasts may increase the likelihood of thoracic vertebral fractures by increasing the mechanical loading of the spine. We examined breast size as a factor associated with prevalent thoracic vertebral fractures, also considering its relationship with thoracic kyphosis and upper back extensor muscle endurance. Using a cross‐sectional study, the design measurements collected were thoracic vertebral fractures (≥20% loss in vertebral body height on lateral radiograph), breast size (bra size converted to an ordinal breast size score), BMD (g/cm^2^ averaged femoral neck, DXA), upper back extensor muscle endurance (isometric chest raise test), body composition (DXA), thoracic kyphosis (radiograph), and upper back pain (numerical rating scale). Correlations and multivariable logistic regression examined relationships between characteristics and their association with vertebral fracture. Participants were 117 healthy postmenopausal women. The 17 (15%) women with ≥1 thoracic vertebral fracture had larger breast size (mean difference [MD]: 2.2 sizes; 95% CI, 0.6 to 3.8 sizes), less upper back extensor muscle endurance (MD: −38.6 s; 95% CI, −62.9 to −14.3 s), and greater thoracic kyphosis (MD: 7.3°; 95% CI, 1.7° to 12.8°) than those without vertebral fracture. There were no between group differences in age, height, weight, and BMD. Breast size (*r* = −0.233, *p* = 0.012) and thoracic kyphosis (*r* = −0.241, *p* = 0.009) correlated negatively with upper back extensor muscle endurance. Breast size was unrelated to thoracic kyphosis (*r* = 0.057, *p* = 0.542). A (final) multivariable model containing breast size (OR 1.85; 95% CI, 1.10 to 3.10) and thoracic kyphosis (OR 2.04; 95%CI, 1.12 to 3.70) explained 18% of the variance in vertebral fracture. Breast size had a significant, but weak relationship with vertebral fracture (*R* ^2^ = 0.10), which was independent of BMD and unrelated to thoracic kyphosis. Further work is needed to confirm larger breast size as a risk factor for vertebral fracture. © 2020 The Authors. *JBMR Plus* published by Wiley Periodicals, Inc. on behalf of American Society for Bone and Mineral Research.

## Introduction

Thoracic vertebral fractures have negative consequences for physical function^(^ 1, 2, 3 ^)^ and can lead to progressive disability and significant healthcare costs.^(^ ^4^ ^)^ Postmenopausal women are at greater risk of vertebral fractures.^(^ ^5^ ^)^ Fracture risk is the function of bone strength and the loads to which it is exposed. Many factors have been associated with fracture risk, with plausible relationships either via effects on bone strength or via loading of the thoracic vertebra.^(^ ^6^ ^)^ Clear mechanisms for other factors that appear to be related are yet to be determined.

One aspect of the female physique that may contribute to the risk of thoracic vertebral fractures that has not been explicitly investigated is breast size. Larger breast size is associated with a more habitually flexed posture, and greater thoracic kyphosis and upper back pain.^(^ 7, 8, 9, 10 ^)^ Breast size also accounts for up to 29% of the variance in trunk muscle activity,^(^ ^9^ ^)^ and increasing breast weight magnifies compressive forces on the thoracic spine.^(^ ^11^ ^)^ It follows that large heavy breasts could heighten the vulnerability of women to vertebral fractures.

In this exploratory study, we examined the relationships between prevalent thoracic vertebral fractures and breast size, thoracic kyphosis, and upper back extensor muscle endurance in healthy postmenopausal women.

## Materials and Methods

Participants were initially recruited for a larger survey‐based study examining relationships between physical characteristics and upper back pain in postmenopausal women. The need for volunteers was advertised via radio, newspaper, and online. Advertising was designed to attract women of all breast sizes with and without upper back pain. To be included in the survey, sample volunteers were required to reside in Australia, to read and understand English, and be aged ≥40 years. Exclusion criteria were past history of breast surgery or thoracic spine surgery; systemic inflammatory conditions; neurodegenerative disorders; any known pathology of the breast, lung, or thoracic spine; or recent or long‐term use of steroid or pain medication. From the 269 participants recruited for the survey, consecutive postmenopausal women who provided their contact details were invited to participate further and undergo objective measures. Women who classified themselves as postmenopausal and reported their last menstrual period was more than 12 months previously were defined as postmenopausal. The target was to have a sample of 100 women: 50 who reported upper back pain and 50 without upper back pain. The study was approved by the Human Research Ethics Committee at Curtin University (RDHS‐267‐15); all participants provided written informed consent.

Data regarding medical history and the presence (yes/no) and severity (numerical rating scale [NRS]) of upper back pain within the previous month were collected in the survey study using an online questionnaire (version June 2016; Qualtrics, Provo, UT, USA). Upper back pain was defined as pain in the spine region above the base of the rib cage and below the neck.

Objective data were collected at a university health clinic by an experienced, female musculoskeletal physiotherapist who had completed over 50 hours of training and practice of the methods used. The physiotherapist was not aware of the individual participant's questionnaire data at the time objective testing was conducted. Participants' height (cm) and weight (kg) were objectively measured and used to calculate BMI (kg/m^2^). Other physical measures included breast size, BMD, body composition, and upper back extensor muscle endurance. The radiographic assessments of thoracic kyphosis and prevalent vertebral fractures were completed at local branches of a large radiological practice. Participants were referred for a single X‐ray after completing all other physical measures.

Breast size was determined using a traditional measure of bra size that included underbust and overbust measures.^(^ ^12^ ^)^ Triplicate measurements of under bust (intraclass correlation coefficient [ICC] 0.999; 95% CI, 0.996 to 0.999) and overbust (ICC 0.881; 95% CI, 0.770 to 0.947) circumference in 20 women aged ≥40 years showed good to excellent reliability. Bra sizes^(^ ^13^ ^)^ were converted into a continuous breast size score (BSS) between 0 to 18 (Supplemental Fig. S1) using a system conceptually similar to sizing breast prostheses following unilateral mastectomy^(^ ^14^ ^)^ that has been used in prior research.^(^ ^15^ ^)^ Using this system, a 1‐cup‐size increase (eg, C to D) on the same band size (under bust, eg, size 12) is equivalent to a 1‐point increase in BSS. Similarly, a one‐band‐size increase (eg, 12 to 14) with no change in cup size is also a 1‐point increase in BSS.

BMD and body composition were assessed using DXA. Scans were performed using a Lunar Prodigy device (Model DPX 8743) with Encore software (GE Healthcare, Little Chalfont, UK). Standard quality assurance tests, including calibration measurement using a phantom spine, were completed daily prior to use according to the manufacturer's guidelines. The average BMD (g/cm^2^) of the left and right femoral necks (FNs) was calculated and used as the measure of BMD. Duplicate scans of 11 women conducted by our operator within a 12‐month period showed excellent intrarater reliability (ICC 0.974; 95% CI, 0.908 to 0.993) and a coefficient of variation of 1.3%.

Body composition was assessed using whole‐body scans with participants in a supine position. Total fat mass (kg) and lean mass (kg) were recorded. All scans were checked immediately after acquisition and later reviewed by a study supervisor with over 20 years of experience.

Upper back extensor muscle endurance was assessed using the isometric chest raise test.^(^ ^16^ ^)^ Participants were positioned prone over a wedge cushion (Lunamumma, VIC, Australia) with their navel level with the highest edge of the cushion. Adjustable straps were used to secure participants' pelvis and feet to the bed. With their arms unsupported and hands at their temples, participants were asked to raise their chest clear of the bed and hold this position for as long as possible.^(^ ^15^ ^)^ A stopwatch was used to measure the time (in seconds) to failure, defined as the point at which the chest touched the bed. Participants who were unable to raise their chest to initiate the test were allocated a time of zero. An upper limit cut‐off time of 300 s was imposed.^(^ ^15^ ^)^ The isometric chest raise test has high reliability (ICC 0.93 to 0.97) and reproducibility (*r* = 0.94 to 0.9.5) when used with women (aged 35 to 49 years) with and without chronic back pain.^(^ ^16^ ^)^

A single lateral X‐ray of each participant was obtained using standardized instructions. Participants were positioned standing with their arms elevated to approximately 90 degrees. X‐ray devices were positioned at a film focus distance of 120 cm with the beam centered on the midthoracic vertebrae. The X‐ray was evaluated by one radiologist (RH) blinded to the aims of the study. Thoracic kyphosis (°) was measured using the four‐segment vertebral centroid global angle method as previously described.^(^ ^17^ ^)^ The midpoints of the upper two (T1, T2) and lowest two (T11, T12) most clearly visible thoracic vertebral bodies were used to determine the vertebral centroid angles using digital software (InteleViewer, Inteleard, Montreal, Canada). Prevalent vertebral fractures were identified as those vertebrae with a 20% reduction in vertebral body height relative to normal adjacent vertebrae.^(^ ^18^ ^)^ The radiologist (RH) completed all assessments without any clinical information about each participant.

## Statistical analysis

A priori sample size calculation indicated that our sample of 117 would be sufficient to detect a minimum change in odds for prevalent vertebral fracture of 0.62 with 80% power and a confidence level of 95%.^(^ ^19^ ^)^

Descriptive summaries were calculated for all participant characteristics and included means and standard deviations for continuous data and frequency distributions for categorical data. The sample was dichotomized into vertebral fracture (participants with ≥1 vertebral fracture) and nil vertebral fracture groups. Independent samples *t* tests or chi‐square analyses were used to compare participant characteristics.

To explore the relationships between the independent variables of breast size, thoracic kyphosis, and upper back extensor muscle endurance, Pearson's correlation coefficients (*r*) were calculated. The strength of relationships were interpreted as weak (*r* ≤ 0.25), fair (*r* = 0.25 to 0.5), moderate (*r* = 0.50 to 0.75), or strong (*r* > 0.75).^(^ ^20^ ^)^ Logistic regression models were used to examine the association between these characteristics and the dependent variable prevalent vertebral fracture (yes/no). Results were summarized using ORs and 95% CIs. All ORs were standardized by calculating them for a 1‐SD change in the variable of interest. Bivariate models were examined prior to conducting a multivariable logistic regression analysis where all independent variables of interest (breast size, thoracic kyphosis, and upper back extensor muscle endurance) were examined together. Bivariate models were tested with and without adjustment for age and BMD. Variations of the multivariable model that were examined included adjustment for BMD in step 1, and the model with and without thoracic kyphosis. Three interaction terms were also explored in the multivariable model (breast size * thoracic kyphosis, breast size * upper back extensor muscle endurance, thoracic kyphosis * upper back extensor muscle endurance). The final and most optimal model was built using backward conditional procedures and contained only those variables making a significant contribution (*p* < 0.05). The assumptions (linearity of independent variables and log odds, absence of multicollinearity and outliers) of the model were checked.

Data were analyzed using SPSS version 24 (SPSS, Inc., Chicago, IL, USA). All hypothesis tests were two‐sided and *p*‐values <0.05 were considered statistically significant.

## Results

**Figure 1:**
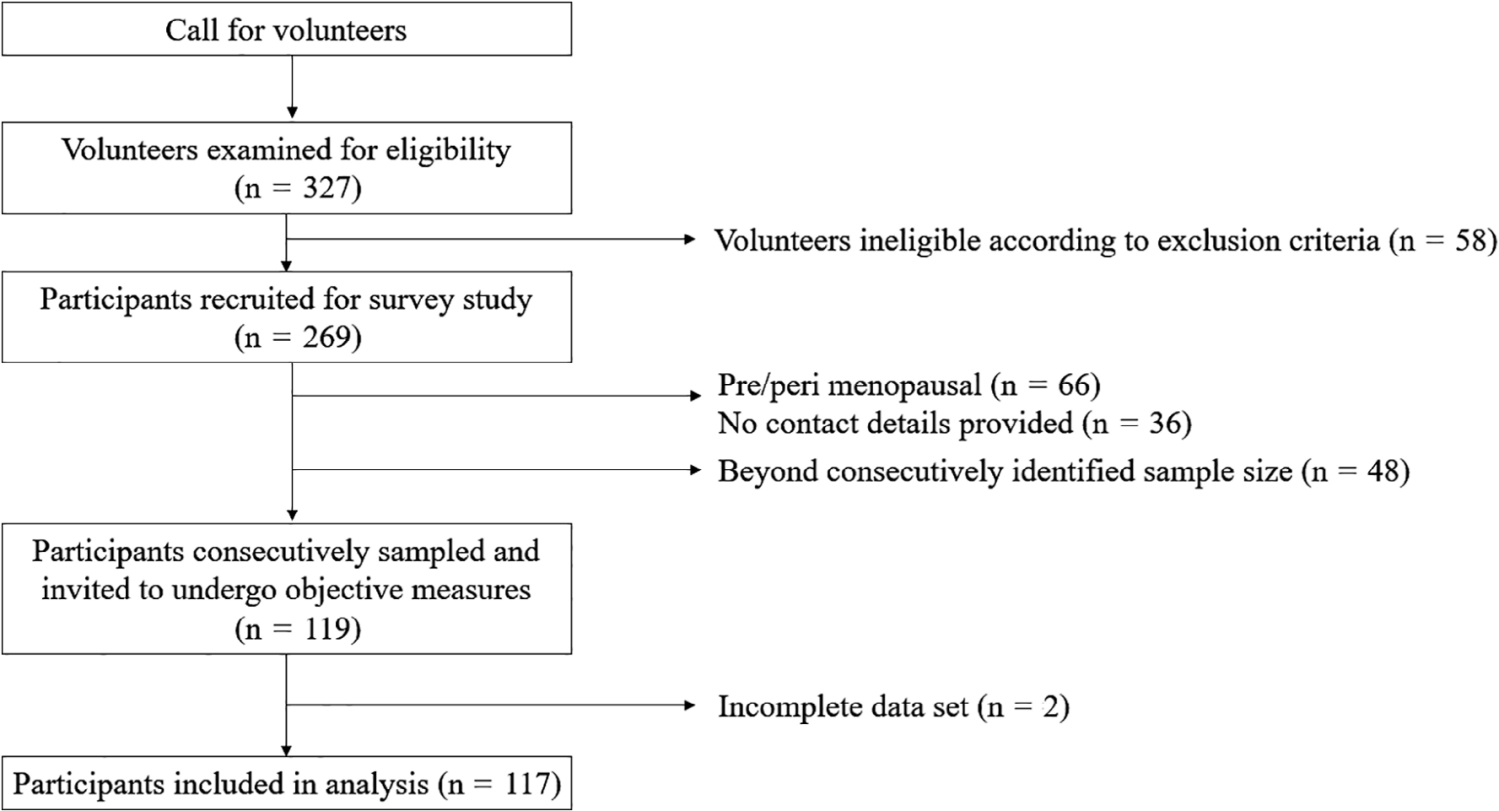
Participant recruitment to the study.

The data of 117 postmenopausal women who had completed all relevant measures were analyzed (Fig. 1). Participant characteristics are summarized in Table 1. Seventeen (15%) participants had radiological evidence of ≥1 vertebral fracture. Two participants (one from each group) had osteoporosis with a left FN *T*‐score of ≤ −2.5. Osteopenia (left FN *T*‐score −2.5 to −1) was identified in 47 (40%) participants, and 6 (13%) from the vertebral fracture group. Vertebral fractures were located in the mid to lower thoracic spine (Table 1). Thirteen (76%) participants in the fracture group reported having upper back pain compared with 47 (47%) participants in the nil fracture group (*p* = 0.025). All participants were able to lift their chest off the bed and three participants reached the upper limit threshold of 300 s when measuring upper back extensor muscle endurance using the isometric chest raise test.

**Table 1:** Descriptive Summary of Sample

Participant characteristics Mean (SD)	Total (*n* = 117)	Nil fracture (*n* = 100)	Fracture (*n* = 17)	MD (95% CI)	*p*‐Value
Age (years)	61.4 (7.0)	61.6 (7.1)	60.4(6.8)	−1.3 (−4.9 to 2.4)	0.496
Height (cm)	161.3 (6.2)	161.2 (6.4)	161.5 (4.6)	0.3 (−3.0 to 3.5)	0.874
Weight (kg)	75.4 (15.3)	74.4 (14.3)	81.1 (19.2)	6.7 (−1.2 to 14.6)	0.095
BMI (kg/m^2^)	29.0 (5.5)	28.6 (5.0)	31.1 (7.5)	2.5 (−1.4 to 6.5)	0.198
Fat mass (kg)	31.9 (10.3)	31.7 (10.0)	33.2 (12.2)**a**	1.6 (−4.0 to 7.1)	0.578
Lean mass (kg)	40.5 (5.8)	40.3 (5.8)	42.4 (5.8)**a**	2.1 (−0.9 to 5.2)	0.170
Breast size (breast size score)	7.9 (2.5)	6.8 (2.8)	8.9 (4.3)	2.2 (0.6 to 3.8)	0.008
BMD (femoral neck) (g/cm^2^)	0.907 (0.113)	0.906 (0.109)^b^	0.909 (0.142)	−0.004 (−0.056 to 0.063)	0.898
BMD Femoral neck (age adjusted *Z*‐score)	0.349 (0.815)	0.366 (0.796)^b^	0.253 (0.937)	−0.113 (−0.538 to 0.313)	0.602
Thoracic kyphosis centroid angle (°)	42.2 (10.9)	41.2 (10.4)	48.4 (12.0)	7.3 (1.7 to 12.8)	0.011
Upper back extensor muscle endurance (s)	97.1 (70.7)	102.7 (73.4)	64.1 (39.2)	−38.6 (−62.9 to −14.3)	0.003
Upper back pain NRS scores	2.3 (2.7)	2.1 (2.8)	3.4 (2.2)	1.3 (−0.1 to 2.7)	0.076
Fracture characteristics (*n* = 17)	Frequency (%)				
Participants with 1 vertebral fracture	11 (64)				
Participants with 2 vertebral fractures	4 (24)				
Participants with 3 vertebral fractures	2 (12)				
Fracture locations (*n* = 25 fractures in 17 participants)	Frequency (%)				
T1 to T5	0 (0)				
T6	2 (8)				
T7	5 (20)				
T8	6 (24)				
T9	1 (4)				
T10	1 (4)				
T11	5 (20)				
T12	5 (20)				

a One missing value.

b Two missing values.

NRS = numerical rating scale; MD = mean difference.

Breast size scores ranged from 2 to 18 (equivalent to Australian bra sizes 10A to 26H). Median (interquartile range [IQR]) underbust and overbust circumferences were 87.0 cm (11.8 cm) and 102 cm (16.0 cm) and the median (IQR) breast size score of 7.0 (4.0), equivalent to a bra size of, for example, 16C. The average equivalent bra size of participants with vertebral fracture was 16DD (BSS = 9). This was two sizes larger than the average equivalent bra size of participants without fracture (16C/BSS = 7). For other equivalent bra sizes, see supplementary Fig. S1.

Participants with prevalent vertebral fracture(s) had significantly larger breasts, less upper back extensor muscle endurance, and greater thoracic kyphosis compared with those without vertebral fracture (Table 1). There were no differences in age, height, weight (total lean or fat), or BMD between the fracture and nil fracture groups (Table 1).

There were weak negative relationships between breast size and upper back extensor muscle endurance (*r* = −0.233, *p* = 0.012), and between thoracic kyphosis and upper back extensor muscle endurance (*r* = −0.241, *p* = 0.009); however, breast size was not correlated to thoracic kyphosis (*r* = 0.057, *p* = 0.542).

Each of the three variables of interest was associated with prevalent vertebral fracture (Table 2). Neither age nor BMD were associated with prevalent vertebral fracture and adjusting for them made little difference to any of these associations.

**Table 2:** Associations Between the Independent Variables of Interest and Prevalent Vertebral Fracture in Bivariate Analysis (Logistic Regression)

Independent variable	*n*		OR (95% CI)	
		Unit of comparison (equivalent to 1 SD)		*p*‐Value
Age (years)	117	+7.0 years	0.83 (0.50–1.40)	0.492
BMD (femoral neck) (g/cm^2^)^a^	115	−0.113 g/cm^2^	0.97 (0.58–1.62)	0.897
Breast size (breast size score)	117	+2.5 BSS sizes	1.89 (1.15–3.11)	0.013
Thoracic kyphosis centroid angle (°)	117	+10.9°	2.11 (1.16–3.83)	0.014
Upper back extensor muscle endurance (s)	117	−70.7 s	2.23 (1.00–4.94)	0.046

a Two missing values because of bilateral total hip prostheses.

BSS = breast size score.

The final and most optimal multivariable model included breast size and thoracic kyphosis (Table 3). This model was statistically significant, χ^2^(2) 12.5, *p* = 0.002, and explained a total of 18% (Nagelkerke *R* ^*2*^) of the variance in vertebral fracture (Table 3). Breast size made a small significant contribution to the model, accounting for 10% of this variance. Upper back extensor muscle endurance did not improve the variance explained by model when included (*p* = 0.085) and BMD, when entered in step 1, was not significant (*p* = 0.665).

**Table 3:** Factors Associated With Prevalent Vertebral Fracture in Multivariable Analysis (Final Model)

Independent variable	*n*		OR (95% CI)	
		Unit of comparison (equivalent to 1 SD)		*p*‐Value
Breast size (BSS)	117	+2.5 BSS sizes	1.85 (1.10–3.10)	0.020
Thoracic kyphosis centroid angle (°)	117	+10.9°	2.04 (1.12–3.70)	0.020

Note. Model was run backward conditional (*p* for inclusion <0.05) with forward conditional confirming the results. BSS = breast size score.

Changes to the model structure (with and without BMD and/or thoracic kyphosis) did not change the contribution made by breast size, which remained significant, but weak with *R* ^2^ consistent at 0.10. The inclusion of thoracic kyphosis in the model had negligible influence on the association between breast size and prevalent vertebral fracture. With thoracic kyphosis excluded from the model, upper back extensor muscle endurance remained nonsignificant.

Interaction terms between breast size and upper back extensor muscle endurance (*p* = 0.815), between breast size and thoracic kyphosis (*p* = 0.234), and between thoracic kyphosis and upper back extensor muscle endurance (*p* = 0.143) were not associated with the outcome prevalent vertebral fracture and the final multivariable model remained virtually unchanged in each case.

## Discussion

In this study, we found that healthy postmenopausal women with larger breast size are more likely to have prevalent vertebral fractures. The relationship between breast size and vertebral fracture, albeit weak, has been identified to be independent of BMD, age, thoracic kyphosis, and upper back extensor muscle endurance.

There could be a biomechanical rationale linking larger breasts to the presence of vertebral fractures, but breast size has not been previously considered as an associated risk factor for prevalent vertebral fractures. Breast size is a physical characteristic that, by increasing the forces acting on the spine,^(^ ^11^ ^)^ may influence the biomechanical loads to which the spine is exposed. As such, it is possible that breast size may be interacting with other factors within the local environment of the vertebral body that affects its integrity.^(^ ^6^ ^)^

Thoracic flexion torques are reported to be up to 5 times greater in women with large breasts compared with women with small breasts.^(^ 8, 21 ^)^ It is possible that with greater thoracic flexion torques there are greater vertebral compression loads,^22^, ^23^^)^ which could increase the risk for vertebral fractures.^(^ ^6^ ^)^ Some previous accounts show that large breasts are associated with greater thoracic kyphosis,^(^ 7, 8 ^)^ an important factor generating greater thoracic flexion torques and increasing vertebral compression loads.^(^ 22, 23 ^)^ These findings provided a rationale for exploring the possibility of a relationship between breast size, thoracic kyphosis, and vertebral fracture.

Although thoracic kyphosis was associated with vertebral fracture, we did not find an association between breast size and thoracic kyphosis. This was an unexpected finding, but with good heterogeneity in terms of breast size and thoracic kyphosis, we have no reason to doubt it. Interestingly, a recently published larger study (*n* = 300) also reported that breast size was unrelated to thoracic kyphosis.^(^ ^21^ ^)^ Given the cross‐sectional design of our study, it is not possible to determine whether thoracic kyphosis contributed to the risk of incident vertebral fractures (detected as prevalent fractures in our sample) or whether kyphosis was a consequence of the prevalent vertebral fractures. As we found no significant interactions suggesting that the relationship between breast size and vertebral fracture depended on the degree of thoracic kyphosis, it appears they are independent relationships in our group of healthy postmenopausal women. Longitudinal studies to determine the temporal relationship between breast size, thoracic kyphosis, and fracture risk are required to determine causality.

BMD was comparable between the fracture and nil fracture groups. In the context of our selection criteria, excluding volunteers with diagnosed osteoporosis or known vertebral fractures (known pathology of the thoracic spine), this finding was less surprising than it would have been in a community‐based sample. On average, the BMD *Z*‐scores (age‐adjusted) in our sample sit slightly above the population‐based mean of zero, and the prevalence of osteoporosis was less than would be expected from epidemiological evidence.^(^ ^24^ ^)^ Of interest, however, despite the exclusion criteria, the prevalence of vertebral fractures in our sample was 15%, which is consistent with rates reported for women aged over 50 years in two large population studies.^(^ 25, 26 ^)^ The lack of association between BMD and prevalent fracture suggests that the vertebral fractures were not specifically a feature of poor vertebral strength. Although we acknowledge that aspects of bone strength not assessed by areal BMD cannot be ruled out in our study, it may be that factors related to vertebral loading provide the biomechanical basis for the relationship between breast size and vertebral fracture.

Upper back extensor muscle endurance was considered in the current study to be a suitable marker of trunk muscular support and was investigated to explore speculation that it has an important role in offsetting the biomechanical burden of larger breasts and in mitigating the progression of thoracic kyphosis and consequent upper back pain.^(^ ^8^ ^)^ Our correlational analysis supports this speculation by showing a significant negative relationship between upper back extensor muscle endurance and breast size and between upper back extensor muscle endurance and thoracic kyphosis.

The endurance capacity of upper back muscles was considered particularly important to a biomechanical relationship involving breast size, thoracic kyphosis, and thoracic vertebral fractures given the high prevalence of slow twitch (type I) fibers in the erector spinae of the upper back, which suggests these are postural muscles responsible for slow and sustained contractions.^(^ ^27^ ^)^ Trunk muscles have been previously discussed as an important feature implicated with the presence of vertebral fractures in postmenopausal women.^(^ 28, 29 ^)^ Highlighting this importance are the losses in size and density of important spine stabilizing muscles that occur more profoundly in women than in men with advancing age.^(^ ^30^ ^)^ Trunk muscles could affect vertebral loading and fracture risk by relating to thoracic kyphosis,^31^, ^32^, ^33^^)^ but it cannot be assumed that declining thoracic musculature with aging alone will increase thoracic kyphosis.^(^ ^34^ ^)^ Upper back extensor muscles with better endurance provide the spine with better stability, creating less loading on the intervertebral joints^(^ ^35^ ^)^ and reducing skeletal and ligamentous strain.^(^ ^36^ ^)^ In the presence of an accentuated thoracic kyphosis, however, the capability of upper back extensor muscles to generate force over time may be affected by the length‐tension relationships of these muscles.^(^ ^6^ ^)^ This decrease in capability may explain the negative relationship we have identified and why vertebral fractures were more likely in women with greater thoracic kyphosis and those with poorer upper back extensor muscle endurance.

Our bivariate analysis findings of an association between upper back extensor muscle endurance and prevalent vertebral fractures is consistent with the protective role accorded to the upper back extensor muscles in previous studies of vertebral fracture.^(^ 28, 33 ^)^ Although the contribution of upper back extensor muscle endurance was not significant in our final multivariable model, it is important to highlight that both breast size and thoracic kyphosis independently reduced the strength of the relationship between upper back extensor muscle endurance and prevalent vertebral fracture. The decreasing OR for upper back extensor muscle endurance in the presence of breast size and thoracic kyphosis within the multivariable model for vertebral fracture perhaps reflects the antagonistic relationship between upper back extensor muscle endurance and each of these variables, respectively. The interplay between these variables would be worthy of consideration in future studies looking at training upper back extensor muscle endurance to reduce the risk of vertebral fractures, particularly in women with large breasts. For the likelihood of vertebral fracture, however, it appears that breast size and thoracic kyphosis are not influenced strongly by upper back extensor muscle endurance.

In this study, we present breast size as a new and novel characteristic associated with vertebral fracture. Women with larger breasts were more likely to have prevalent vertebral fractures with odds that are comparable to other characteristics previously reported.^(^ 37, 38 ^)^ Our findings suggest that, unrelated to BMD and thoracic kyphosis, vertebral facture may turn out to be an important clinical consequence of large breasts, which may account for why upper back pain is more common in women with large breasts.^(^ 8, 10, 39 ^)^ Back pain has previously been related to the severity and number of prevalent^(^ ^3^ ^)^ and incident^(^ ^2^ ^)^ vertebral fractures in women with osteoporosis. Our findings indicate that upper back pain was more likely in participants with vertebral fractures compared with those without, but that the severity of this pain was not significantly different between groups. Pain severity was described by the fracture group as being mild (NRS <4)^(^ ^40^ ^)^ however, it is possible that owing to the small size of the fracture group, there was not a sufficient spread across the range of possible severity scores. This may have made it difficult for us to find a significant difference in upper back pain severity between groups. Future research might look at other samples of women to establish if vertebral fractures are related to the symptomatic burden in those with large breasts.

One limitation of our study is that the definition of vertebral fracture we used is only one of several that are available;^(^ 18, 41, 42, 43 ^)^ other definitions may have yielded different results. The precise measurement of intact breast size is notoriously difficult because of the complex and varied morphology of the breast.^(^ ^44^ ^)^ To date, there is no perfectly valid noninvasive method for measuring breast size, and this is a challenge for all nonsurgical studies in this clinical area. For the purposes of this study, we selected a breast size scoring method that allowed us to rank the breast size of our participants. This was sufficient to identify the relationship between breast size and prevalent vertebral fracture, but does not enable examination of precise volumes that may or may not be problematic.

In conclusion, the relationship between breast size and vertebral fracture is identified in this study to be weak. Breast size accounted for only a small proportion of explained variance in prevalent vertebral fracture. Other physical characteristics and established risk factors,^(^ 37, 38 ^)^ which have not been assessed in this study, are likely to explain the remaining variance. Consequently, breast size needs to be further examined alongside these other characteristics and risk factors to confirm breast size as a potential risk factor for vertebral fracture.

## Disclosures

The authors have nothing to disclose.

## Supporting information


**Supplementary Material Fig. S1**Breast size score conversion chart.
