# Supplementary material for: Is Breast Size Related to Prevalent Thoracic Vertebral Fracture? A Cross‐Sectional Study
Source: JBMR Plus. 2020 May 19;4(7):e10371. doi: 10.1002/jbm4.10371 (PMC7340439; doi:10.1002/jbm4.10371)
Supplement: Supplementary file 1 — Supplementary Material Fig. S1Breast size score conversion chart. [file JBM4-4-e10371-s001.pdf]

Supplemental Material

		**Band size** (under-bust circumference, cm)									
	**Cup size**	**8** (63-67)	**10** (68-72)	**12** (73-77)	**14** (78-82)	**16** (83-87)	**18** (88-92)	**20** (93-97)	**22** (98-102)	**24** (103-107)	**26** (108-112)
**Breast size score** (over-bust circumference, cm)	**AA**	**0** (75-77)	**1** (80-82)	**2** (85-87)	**3** (90-92)	**4** (95-97)	**5** (100-102)	**6** (105-107)	**7** (110-112)	**8** (115-117)	**9** (120-122)
	**A**	**1** (77-79)	**2** (82-84)	**3** (87-89)	**4** (92-94)	**5** (97-99)	**6** (102-104)	**7** (107-109)	**8** (112-114)	**9** (117-119)	**10** (122-124)
	**B**	**2** (79-81)	**3** (84-86)	**4** (89-91)	**5** (94-96)	**6** (99-101)	**7** (104-106)	**8** (109-111)	**9** (114-116)	**10** (119-121)	**11** (124-126)
	**C**	**3** (81-83)	**4** (86-88)	**5** (91-93)	**6** (96-98)	**7** (101-103)	**8** (106-108)	**9** (111-113)	**10** (116-118)	**11** (121-123)	**12** (126-128)
	**D**	**4** (83-85)	**5** (88-90)	**6** (93-95)	**7** (98-100)	**8** (103-105)	**9** (108-110)	**10** (113-115)	**11** (118-120)	**12** (123-125)	**13** (128-130)
	**DD**	**5** (85-87)	**6** (90-92)	**7** (95-97)	**8** (100-102)	**9** (105-107)	**10** (110-112)	**11** (115-117)	**12** (120-122)	**13** (125-127)	**14** (130-132)
	**E**	**6** (87-89)	**7** (92-94)	**8** (97-99)	**9** (102-104)	**10** (107-109)	**11** (112-114)	**12** (117-119)	**13** (122-124)	**14** (127-129)	**15** (132-134)
	**F**	**7** (89-91)	**8** (94-96)	**9** (99-101)	**10** (104-106)	**11** (109-111)	**12** (114-116)	**13** (119-121)	**14** (124-126)	**15** (129-131)	**16** (134-136)
	**G**	**8** (91-93)	**9** (96-98)	**10** (101-103)	**11** (106-108)	**12** (111-113)	**13** (116-118)	**14** (121-123)	**15** (126-128)	**16** (131-133)	**17** (136-138)
	**H**	**9** (93-95)	**10** (98-100)	**11** (103-105)	**12** (108-110)	**13** (113-115)	**14** (118-120)	**15** (123-125)	**16** (128-130)	**17** (133-135)	**18** (138-140)

Supplemental material. Breast Size Score (BSS) Conversion Chart[a]

[a] To determine a breast size score first identify the correct bra size (band and cup size). Establish the correct band size by measuring around the body, directly below the bust (under-bust circumference) and the correct cup size by measuring across the fullest part of the breasts whilst wearing a bra (over-bust circumference)[(13)]. Use the top row of the table to select the band size, this increases from left to right. Then use the first column of the table to select the cup size, this increases from top to bottom. Track down and across to find the table cell where these two selections intersect. Breast size score is shown in bolded text.
